# Metabolomic analysis to discriminate drug-induced liver injury (DILI) phenotypes

**DOI:** 10.1007/s00204-021-03114-z

**Published:** 2021-07-17

**Authors:** Guillermo Quintás, Teresa Martínez-Sena, Isabel Conde, Eugenia Pareja Ibars, Jos Kleinjans, José V. Castell

**Affiliations:** 1 Health and Biomedicine, Leitat Technological Center, Valencia, Spain; 2 Unidad de Hepatología Experimental, Health Research Institute La Fe, Valencia, Spain; 3 Departamento de Bioquímica y Biología Molecular, Universidad de Valencia, Valencia, Spain; 4 Unidad Analítica, Health Research Institute La Fe, Valencia, Spain; 5 Clinical Hepatotoxicity Unit, University and Politechnic Hospital La Fe, Valencia, Spain; 6 Servicio de Cirugía General y Aparato Digestivo, Hospital Universitario Dr. Peset, Valencia, Spain; 7 Centro de Investigación Biomédica en Red de Enfermedades Hepáticas y Digestivas (CIBERehd), Instituto de Salud Carlos III, Madrid, Spain; 8 Department of Toxicogenomics, Maastricht University, Maastricht, The Netherlands

**Keywords:** Metabolomics, Hepatotoxicity, Drug liver toxicity, DILI, Drug-induced liver injury, Biomarkers

## Abstract

Drug-induced liver injury (DILI) is an adverse toxic hepatic clinical reaction associated to the administration of a drug that can occur both at early clinical stages of drug development, as well after normal clinical usage of approved drugs. Because of its unpredictability and clinical relevance, it is of medical concern. Three DILI phenotypes (hepatocellular, cholestatic, and mixed) are currently recognized, based on serum alanine aminotransferase (ALT) and alkaline phosphatase (ALP) values. However, this classification lacks accuracy to distinguish among the many intermediate mixed types, or even to estimate the magnitude and progression of the injury. It was found desirable to have additional elements for better evaluation criteria of DILI. With this aim, we have examined the serum metabolomic changes occurring in 79 DILI patients recruited and monitored using established clinical criteria, along the course of the disease and until recovery. Results revealed that free and conjugated bile acids, and glycerophospholipids were among the most relevant metabolite classes for DILI phenotype characterization. Using an ensemble of PLS–DA models, metabolomic information was integrated into a ternary diagram to display the disease phenotype, the severity of the liver damage, and its progression. The modeling implemented and the use of such compiled information in an easily understandable and visual manner facilitates a straightforward DILI phenotyping and allow to monitor its progression and recovery prediction, usefully complementing the concise information drawn out by the ALT and ALP classification.

**Supplementary Information:**

The online version contains supplementary material available at 10.1007/s00204-021-03114-z.

## Introduction

Drug-induced liver injury (DILI) is a serious toxic event that can occur in the course of early drug development as well upon clinical usage or over-the-counter drug self-consumption. It is among the most frequent manifestation of liver toxicity, and the most cited reason for drug development discontinuation and withdrawal from the market. As such, it is of public health interest and growing concern for poly-medicated patients in western societies. DILI represents the leading cause of acute liver failure in Europe and the United States with an estimated incidence varying from 2.4 to 19 per 100,000 patient-years, and is the main cause for urgent liver transplantation due to acute liver failure (Björnsson et al. 2013; Sgro et al. 2002; Abajo et al. 2004). Besides its intrinsic morbidity, the number of DILI events is raising in parallel to the introduction of new drugs, the increased life expectancy, poly-medication in elderly people, and the widespread use of self-prescribed complementary dietetic or herbal products (Lin et al. 2019). DILI events are classified according to their clinical and pathological presentations as cholestatic, hepatocellular, or mixed types (i.e., sharing cholestatic and hepatocellular features) (Zimmerman 2000). Hepatocellular toxic reactions are the most straightforward identifiable and abrupt onset type of DILI reactions, constituting up to 90% of all cases (Larrey 2000). These reactions are characterized by liver cell necrosis and a concomitant inflammation, mild bile stasis, and markedly elevated levels of serum alanine aminotransferase (ALT) and aspartate aminotransferase (AST), and rather moderate elevations of alkaline phosphatase (ALP) and gamma-glutamyl transferase (GGT). Cholestatic DILI resembles bile duct mechanical obstruction (cholelithiasis), with bile flux stasis, jaundice, portal inflammation, proliferation or injury of bile ducts, ALP and GGT levels substantially elevated, while ALT and AST levels remain minimally elevated. The mixed-type injury share both characteristics and is characterized by elevations in both serum ALT/AST ratio (ALT/AST) and ALP.

The identification of the different phenotypes of DILI by clinical biochemistry end/points relies on the, so-called “*R*-score” defined as

$$R{\text{-score}} = ~\frac{{{{\left[ {{\text{ALT}}} \right]} \mathord{\left/ {\vphantom {{\left[ {{\text{ALT}}} \right]} {\left[ {{\text{ALT}}} \right]_{{{\text{UNL}}}} }}} \right. \kern-\nulldelimiterspace} {\left[ {{\text{ALT}}} \right]_{{{\text{UNL}}}} }}}}{{{{\left[ {{\text{ALP}}} \right]} \mathord{\left/ {\vphantom {{\left[ {{\text{ALP}}} \right]} {[{\text{ALP}}]_{{{\text{UNL}}}} }}} \right. \kern-\nulldelimiterspace} {[{\text{ALP}}]_{{{\text{UNL}}}} }}}},$$

where [ALP] and [ALT] are the patient’s ALP and ALT serum activities and [ALP]_UNL_ and [ALT]_UNL_ are the average upper normal limits. The *R*-score ratio is used as a first approach to the clinical characterization of DILI. *R*-scores > 5 indicates the predominance of hepatocellular transaminases over ductal alkaline phosphatases, and denotes principally hepatocellular liver injury; 2 < *R*-scores < 5 denote mixed liver injury; and *R*-scores < 2 are indicative of the predominance of ductal over hepatocellular enzymes, and hence, cholestatic liver injury. Minor differences in how the *R*-score is calculated also leads to discrepancies in defining the phenotype of liver injury for a given patient. Thus, whereas some clinicians use the enzyme values from the first analytical test showing elevations above normal to calculate the *R*-score, others use peak values in the course of DILI disease, leading to *R*-scores that may significantly differ. In addition, uncertainties in assessing DILI diagnosis occur, because these analytical parameters are not specific of DILI. Currently, we lack specific analytical biomarkers that could be unequivocally useful for early detection, diagnosis, monitoring, and prognosis of DILI. ALT continues to be recognized and recommended as best to identify hepatocellular DILI, while jaundice is a clear indicator of cholestatic DILI (FDA 2009). Nevertheless, the physician’s evaluation of patients and the use of attrition scales (García-Cortés et al. 2011; Andrade et al. 2019; Maria and Victorino 1997) remain cornerstones to diagnosis.

The use of the *R*-score for diagnosis also presents important limitations when certain mechanisms of toxicity are involved (García-Cortés et al. 2011). The inhibition of the mitochondrial respiratory chain at early stages, for instance, although hepatocellular DILI in nature is neither accompanied by elevated ALT nor ALP values (Russmann et al. 2009). Increased ALT and AST serum levels can also be typical of muscle and cardiac damage, respectively, evidencing a limited tissue specificity (Yang et al. 2014). In addition, ALT, ALP, and AST are not aetiology specific and a basal alterations could be present in case of previous liver diseases (e.g., viral, alcoholic and non-alcoholic steatohepatitis, NASH) (Watkins 2013). The major degree of uncertainty occurs in the mixed-type DILI, where the levels of liver enzymes may correlate poorly with histological patterns and lesion severity (Devarbhavi 2012). On the other hand, elevated levels of ALT that occur during treatment with a potential hepatotoxic drug may return to normal levels upon continuous exposure, despite cell dysfunction continues to evolve (Watkins 2013). Furthermore, the half-life of transaminases is too long biasing the dynamic monitoring of the liver metabolic status, the type of toxic liver injury may change during the course of the illness, and a drug is not always associated to a particular damage pattern (Aithal et al. 2011).

Recent research has identified DILI diagnostic (e.g., protein derived acetaminophen (APAP)-cysteine, for APAP overdose), predictive (e.g., genetic associations), prognostic (e.g., miR-122, high mobility group box 1 (HMGB1) protein, Keratin-18), and mechanistic biomarkers (e.g., HMGB1, Keratin-18, glutamate dehydrogenase (GLDH), mitochondrial DNA or nuclear fragments) (McGill and Jaeschke 2019). In spite of that, major advances are still needed for accurate DILI diagnosis and to translate the biochemical information to decision making in clinical practice.

We have approached this problem by examining any relevant metabolic changes that, occurring in the liver in the course of a DILI event, are reflected in the patient’s sera as well. We monitored these changes in the different DILI types until the patient’s recovery, with the hope of identifying characteristic metabolic signatures of the different DILI phenotypes, as compared to the recovered status. Metabolomics is recognized as a useful phenotyping tool for the disclosure of dysregulated metabolic pathways in cells and tissues and so, for the analysis of disease and treatment responses. The metabolome is considered to provide a global and direct readout of the dynamic biochemical status of a biological system and has been increasingly applied to the study of liver diseases, such as xenobiotic hepatotoxicity, non-alcoholic fatty liver disease (NAFLD), steatosis, fibrosis, cirrhosis, hepatocellular carcinoma, and cholangiocarcinoma (Cañaveras et al. 2016; Araújo et al. 2017; Mattes et al. 2014; Robles-Díaz et al. 2016). Despite some preliminary exploratory research (Tang and Xu 2014; García-Cañaveras 2015; O’Connell and Watkins 2010; Iruzubieta et al. 2015) its use still remains largely unexplored in the study of liver hepatotoxicity and DILI. Assuming that hepatocyte endo-metabolome is likely to be reflected in the liver exo-metabolome to a certain extent, our aim was to identify metabolic changes in sera of DILI patients that reflect the type and extent of any DILI event, to be then used for diagnosing and monitoring DILI progression over time. To this aim, a longitudinal observational clinical study was designed to enable the metabolomic analysis of serum samples from cholestatic, hepatocellular, and mixed DILI patients over time. Results showed that the metabolic profiles of hepatocellular and cholestatic DILI, and recovered patients displayed significant differences, particularly in the bile acids and lipid profiles. Based on the singularities detected, we constructed a set of binary classification models to facilitate the identification of the three different DILI phenotypes, and to generate ternary diagrams that enable a visual and straightforward interpretation of the disease outcomes for the monitoring its progression over time.

## Materials and methods

### Compliance with ethical standards

The present study was approved by the Ethics Committee for Biomedical Research of the Instituto de Investigación Sanitaria, Hospital Universitario y Politécnico La Fe (Valencia, Spain) (approval Nr. 2012/0452) and was conducted in accordance with the relevant guidelines, good clinical practices and legal and ethical regulations. All patients gave written informed consent prior to participate in the clinical study.

### Clinical study: patients

A total of 79 patients that had been referred to the Clinical Hepatotoxicity Unit between 2013 and 2018 for DILI evaluation, agreed to participate and gave written informed consent to participate in the study. DILI diagnosis was established following international criteria of causality involving: a compatible clinical history and standard analytical results, an adequate chronological relationship, the exclusion of other causes (e.g., alcoholism, viral, metabolic, genetic, tumour, autoimmune, biliary diseases), consumption of a drug with a known hepatotoxic potential, and an elevated score in attrition scales of causality (CIOMS/RUCAM > 6) (Danan and Benichou 1993). Based on the CIOMS/RUCAM score (García-Cortés et al. 2011; Benichou et al. 1993), episodes classified as defined, possible or probable (score 6 or higher) were included in this study. Reference diagnosis and classification of the type of hepatic damage into hepatocellular, cholestatic or mixed-type DILI was made by expert clinicians. Patients were classified as cholestatic DILI if ALP ≥ 147 unit/L and *R*-score < 2, as hepatocellular DILI if ALT ≥ 56 unit/L and *R*-score ≥ 5, mixed DILI when 2 < *R*-score < 5, and ‘recovered’ if ALT < 56 unit/L and ALP < 147 unit /L and absence of any clinical or analytical sign of disease. Timing of blood sampling during patient follow-up was selected to match the scheduled clinical monitoring visits. Therefore, the number of samples collected from each patient varied depending on their clinical follow-up and prompt recovery. Blood samples were collected into BD Vacutainer^®^ SST™ II Advance Tubes (BD Biosciences, Spain). After collection, the blood was allowed to clot at room temperature for 15–30 min, followed by centrifugation at 1500×*g* for 10 min in a refrigerated centrifuge at 6 °C. The resulting supernatant was immediately transferred into 100 µL aliquots in clean polypropylene tubes and stored at − 80 °C until analysis. For each patient, gender, age, and standard liver function indicators (ALT, GGT, ALP, total bilirubin, and albumin), and other current variables reflecting liver function were recorded. Altogether, 79 DILI patients were recruited the number of samples collected from each one of them varied between 1 and 9. A total of 283 serum samples were collected and analysed. Among them, 34 were collected from patients showing hepatocellular DILI, 80 cholestasic DILI, 54 mixed DILI, and 115 samples were collected from clinically recovered patients (see Table 1).

**Table 1:** Clinical and demographic data of the samples collected from DILI patients, included in the study, classified according to clinical variables

	Cholestasic	Mixed type	Hepatocellular (HepC)	Recovered	Chol. vs. HepC	Chol. vs. Mixed	Mixed vs. HepC
Samples (*n*)	80	54	34	115	NA	NA	NA
*R*-score	0.8 ± 0.4 [0.2–2.0]	2.5 ± 0.9 [1.3–4.8]	15 ± 13 [5.0–71.5]	0.9 ± 0.5 [0.2–2.5]	< 0.0001^†^	< 0.0001^†^	< 0.0001^†^
ALT	103 ± 105 [13–675]	133 ± 94 [56–438]	744 ± 645 [125–2341]	28 ± 12 [7–54]	< 0.0001^†^	0.09^†^	< 0.0001^†^
ALP	341 ± 280 [148–1617]	138 ± 83 [53–487]	128 ± 66 [60–413]	87 ± 27 [36–145]	< 0.0001^†^	< 0.0001^†^	0.5^†^
AST	74 ± 62 [11–374]	82 ± 75 [25–460]	482 ± 578 [47–2237]	29 ± 14 [12–139]	< 0.0001^†^	0.5^†^	0.0003^†^
GGT	472 ± 616 [10–3268]	189 ± 207 [27–1068]	249 ± 189 [25–895]	61 ± 60 [8–298]	0.004^†^	0.0002^†^	0.2^†^
Total bilirubin	7 ± 10 [0.2–56.8]	2 ± 5 [0.3–36.1]	8 ± 14 [0.4–47.6]	0.7 ± 0.6 [0.2–4.0]	0.7^†^	0.0003^†^	0.02^†^
Albumin	3.9 ± 0.6 [2.9–4.9]	4.1 ± 0.5 [2.8–5.1]	3.9 ± 0.6 [2.4–4.7]	4.3 ± 0.4 [3.2–5.2]	0.9^†^	0.01^†^	0.06^†^
Tryglycerides	122 ± 127 [0–459]	115 ± 127 [49–748]	34 ± 43 [0–205]	122 ± 72 [12–334]	0.0003^†^	0.0004^†^	0.9^†^
Cholesterol	289 ± 262 [50–1621]	207 ± 44 [80–321]	166 ± 73 [25–297]	198 ± 53 [32–356]	0.0005^†^	0.01^†^	0.008^†^
Glucose	91 ± 16 [54–157]	94 ± 30 [64–232]	99 ± 35 [63–249]	94 ± 21 [61–205]	0.2^†^	0.5^†^	0.5^†^
Creatinine	0.9 ± 0.4 [0.4–2.29]	0.7 ± 0.2 [0.19–1.79]	0.9 ± 0.9 [0.3–5.6]	0.8 ± 0.4 [0.21–3.18]	0.8^†^	0.005^†^	0.2^†^
Age	49 ± 21 [9–90]	46 ± 17 [9–78]	46 ± 18 [15–77]	51 ± 17 [20–79]	0.5^†^	0.4^†^	0.9^†^
BMI	24 ± 3 [18–28]	25 ± 3 [17–35]	25 ± 5 [17–34]	25 ± 3 [19–32]	0.1^†^	0.2^†^	0.6^†^
Sex (male/female)	(46/34)	(24/30)	(16/18)	(47/68)	0.9^‡^	0.9^‡^	0.9^‡^

Values within box brackets represent the range

*Chol.* cholestatic DILI, *Mixed* mixed DILI, *HepC.* hepatocellular DILI, *ALT* alanine aminotransferase, *AST* aspartate aminotransferase, *ALP* alkaline phosphatase, *GGT* gamma-glutamyl transferase

^†^Comparison of mean values: *t* test *p* value (unequal variances)

^‡^Comparison of proportions: *N* − 1 Chi-squared test *p* value

### Standards and reagents

Liquid chromatography–mass spectrometry (LC–MS) grade acetonitrile (CH_3_CN) and methanol (CH_3_OH) were obtained from Scharlau (Barcelona, Spain), and formic acid (HCOOH, ≥ 95%) from Sigma-Aldrich Química SL (Madrid, Spain). Ultra-pure water was generated employing a Milli-Q Integral Water Purification System from Merck Millipore (Darmstadt, Germany). Internal standards phenylalanine-D_5_, tryptophan-D_5,_ and caffeine-D_9_ were purchased from C/D/N Isotopes Inc. (Quebec, Canada).

### Sample preparation

A 100 μL sample of the serum fraction were thaw at room temperature. Then, 300 μL of cold (4 °C) CH_3_OH was added for protein precipitation. The sample was homogenized (Vortex shaker, 10 s) and centrifuged at 15000×*g* (10 min, 4 °C). Then, 300 μL of the supernatant was collected and evaporated to dryness under vacuum at 25 °C. The residue was reconstituted in 150 μL of a 1 μM internal standard solution containing phenylalanine-D_5_, tryptophan-D_5_ and caffeine-D_9_ in H_2_O:CH_3_CN (98:2, 0.1% v/v HCOOH).

### Metabolomic analysis

Metabolomic analysis was performed on an Agilent 1290 Infinity ultraperformance liquid chromatograph (UPLC) using a Kinetex C_18_ (100 × 2.1 mm, 1.7 µM) column (Phenomenex, Torrance, USA). Autosampler and column temperatures were set to 4 °C and 55 °C, respectively, and the injection volume was 4 µL. Gradient elution was performed at a flow rate of 400 µL/min as follows: initial conditions of 98% of mobile phase A (H_2_O, 0.1% v/v HCOOH), held for 0.5 min, followed by a linear gradient from 2 to 20% of mobile phase B (CH_3_CN, 0.1% v/v HCOOH) in 4 min and from 20 to 95% B in 4 min. 95% B was held for 1 min and then, a 0.25 min gradient was used to return to the initial conditions, which were held for 2.8 min. Full scan MS data from 70 to 1200 *m*/*z* was collected on an iFunnel quadrupole time of flight (QTOF) Agilent 6550 spectrometer (Agilent Technologies, CA, USA). Samples were analyzed using positive and negative electrospray ionization (ESI) in separate batches. The following ESI parameters were selected: gas temperature (T), 200 °C; drying gas, 14 L/min; nebulizer, 37 psig; sheath gas T, 350 °C; sheath gas flow, 11 L/min. MS spectra recalibration during the analysis was carried out introducing a reference standard into the source via a reference sprayer valve and using the 149.02332 *(*phthalic anhydride), 121.050873 (purine) and 922.009798 (HP-0921) *m/z* in ESI+, as well as 119.036 (purine) and 980.0163 (HP-0921, [M–H + CH_3_COOH]^−^) *m/z* in ESI−, as references.

The analysis of the full sample set was split into two batches to minimize potential drifts in the UPLC–MS system response during the analysis of a large number of samples arising from system contamination. Batch 1 included the analysis of QC replicates (QC1) analysed every eight samples, and two blanks at the end of the sequence. QCs were prepared as a pool of the processed samples included each batch. Batch 2 included the analysis of QC replicates (QC2) analyzed every ten samples, 15 randomly selected samples analyzed in batch 1 for between-batch normalization, and six blanks at the end of the sequence. A set of 10 QCs was injected at the beginning of each batch for system conditioning. Data obtained during column conditioning was excluded from analysis. MS/MS data acquisition for metabolite annotation was carried out using a collision energy set to 25 V, and with automated selection of three precursor ions per cycle and an exclusion window of 0.25 min after two consecutive selections of the same precursor. The QC was repeatedly analyzed using an auto MS/MS method with the following inclusion *m*/*z* precursor ranges: 70–200, 200–350, 350–500, 500–650, 650–800, 800–950, 950–1100, and 1100–1200 Da using a rate of 3 spectra/s in the extended dynamic range mode (2 GHz).

### Peak table generation and batch effect correction

Peak detection, integration, deconvolution and alignment were carried out for each batch separately using XCMS (Smith et al. 2006) in R 3.2.1. The *centWave* method was used for peak detection with the following parameters: mass accuracy = 12 ppm, peak width = (3, 6), snthresh = 6 and prefilter = (3, 10000). A minimum difference in *m/z* of 7.5 mDa was selected for overlapping peaks. Intensity weighted *m/z* values of each feature were calculated using the *wMean* function. Peak limits used for integration were found through descent on the Mexican hat filtered data. Matching peaks across samples was performed using the *nearest* method with mz-retention time (RT) balance of 2, RT tolerance of 3 s and kNN = 2. Missing data points were filled by reintegrating the raw data files in the regions of the missing peaks using the *fillPeaks* method. Peak integration and alignment accuracies were assessed by comparing automated and manual integration results for internal standards and endogenous metabolites, obtaining linear correlation coefficients higher than 0.99.

Within batch effect correction was carried out using the non-parametric QC–SVRC approach employing a Radial Basis Function kernel using a pre-selection of C and optimization of $\varepsilon$ and $\gamma$ using a grid search, leave-one-out cross validation and the RMSECV as target function (Kuligowski et al. 2015; Sánchez-Illana et al. 2018a). C was selected for each LC–MS feature as the median value of the intensities observed in QC replicates. The $\varepsilon$ search range was selected to match the expected instrumental precision (2.5–8% of the median value of the intensities observed for the whole set of QC replicates). The $\gamma$ search interval selected was [1, 10^4^]. LC–MS features with D-ratio* > 20% after within-batch effect correction were removed from analysis (Broadhurst et al. 2018). Between-batch effect correction was carried out using replicated samples across batches by scaling the intensity of each metabolic feature in each sample. The scaling factor was calculated as the ratio between the median intensity in the batch 2 and the median intensity across batches (Sánchez-Illana et al. 2018b). Finally, samples replicated across batches were excluded form batch 1, and samples for which the values of ALP or ALT were not available (17 in total) were excluded from further analysis. This clean up step left two data sets, X_ESI+_ (283 × 4306) and X_ESI−_ (283 × 5016), where each row represents a chromatogram and each column an LC–MS feature, from the analysis of samples collected from 79 patients. LC–MS features were also excluded if the maximum peak area value in blanks multiplied by 10 was larger than the median value in samples, and those annotated as drug metabolites or food components. Metabolite annotation was carried out based on MS/MS data using the Human Metabolome Database (http://www.hmdb.ca) and METLIN (http://www.metlin.scripps.edu) databases, and LipiDex (Hutchins et al. 2018) as described elsewhere (Ten-Doménech et al. 2020) with 0.015 Da or 20 ppm accuracy. Information regarding classes and subclasses of the metabolites was downloaded from the HMDB (http://www.hmdb.ca) and automatically incorporated to the annotation. Further data analysis included 283 samples and 686 annotated ESI ± LC–MS features with median intensity values in at least one of the groups (hepatocellular DILI, cholestatic DILI, mixed DILI or recovered patients) > 15000 AU (see Supplementary Information, SI). Figure SI1 shows the scores of two-components principal component analysis (PCA) models explaining 32% and 43% of the data variation in the ESI+ and ESI− data sets, respectively. The relative position in the scores plot of the QCs (Fig. SI1, top), and the clustering of the samples analysed in both batches (Fig. SI1, bottom), supported the instrumental stability and accuracy of the batch correction algorithm.

### Software and analysis

*t* tests assessed the null hypothesis that the data of two groups (e.g., cholestatic DILI vs. recovered patients) came from independent random samples with equal means with unknown and unequal variances. LC–MS features with *t* test FDR-adjusted *p* values < 0.05 were selected as significantly altered (Benjamini and Hochberg 1995).

**Fig. 1:**
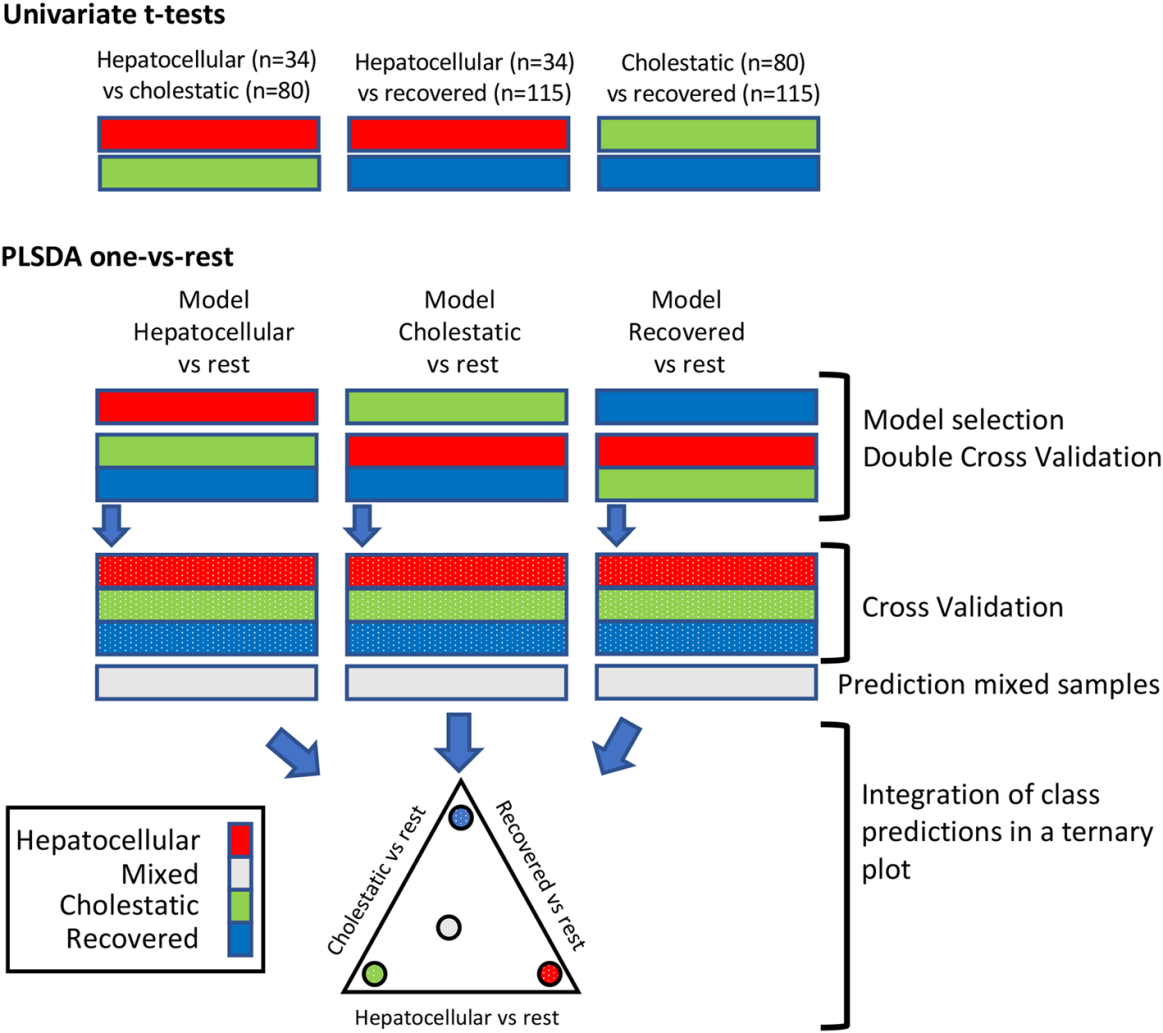
Schematic workflow of data analysis and modelling strategy

Multivariate supervised analysis was carried out by partial least squares–discriminant analysis (PLS–DA). PLS aims to build a linear multivariate model to relate two data matrices *X* and *y* (i.e., the metabolomic data and response variable, which codes for class membership as follows: 1 for the members of one class, 0 (or − 1) for members of the other class) (Wold et al. 2001). Double cross validation (2CV) (Smit et al. 2007) was selected to estimate the out-of-sample PLS–DA prediction error using subjectwise CV. The number of latent variables (LVs) used for each inner PLS model was selected using the sample classification CV-accuracy as target function. PCA, PLS–DA, and univariate analysis (*t* test) were carried out in MATLAB 2017b (Mathworks Inc., Natick, MA, USA) using in-house written scripts and the PLS Toolbox 8.7 (Eigenvector Research Inc., Wenatchee, USA). Ternary plots were build using the *ternaryc* MATLAB function available in FileExchange (http://www.mathworks.com/matlabcentral). SVR models were carried out in MATLAB using the LIBSVM library (Chang and Lin 2011). Raw data conversion into suitable formats to support metabolite annotation was carried out using ProteoWizard (http://proteowizard.sourceforge.net/). LipiDex (Hutchins et al. 2018) was used for metabolite annotation by matching the measured MS/MS spectra to an *in-silico* generated library (LipidBlast) (Kind et al. 2013). A workflow of the data analysis and the strategy to generate the ternary diagrams is depicted in Fig. 1.

## Results and discussion

### Data overview and strategy

Table 1 summarizes the most relevant clinical information concerning the patients and sera samples that were investigated in this study, stratified according to their classification as cholestatic (*n* = 80), hepatocellular (*n* = 34), mixed (*n* = 54) DILI, or as recovered patients (*n* = 115) using the *R* index, the values of AST and ALT and other clinical features. As expected, ALT, ALP, AST, and GGT were differentially expressed between cholestatic and hepatocellular patients, and their distributions showed a significant overlap with the mixed DILI patients. No statistically significant differences (*p* values > 0.05) were found among the distributions of total bilirubin, albumin, glucose, creatinine, age, body mass index, and sex between cholestatic and hepatocellular DILI patients in this study. In addition, significant differences (*p* values < 0.05) were found between the distributions of total bilirubin and cholesterol between the mixed DILI patients and the cholestatic and hepatocellular DILI groups of patients. Figure SI2 summarizes the main subclasses of the LC–MS features annotated in the joint ESI ± data set obtained from patient’s sera after data pre-processing and clean-up. The sub-classes with the largest numbers of annotated LC–MS features were steroids and steroid derivatives, glycerophospholipids, carboxylic acids and derivatives, prenol lipids, fatty acyls, indoles and derivatives, organooxygen compounds, and imidazopyrimidines, accounting for 75% (699) of the initially annotated features.

The overall strategy to generate the discriminant model is depicted in Fig. 1. After an initial data overview by PCA, it consisted in pairwise comparison of metabolomic features of hepatocellular vs. cholestasic DILI, hepatocellular DILI vs. recovered and cholestasic DILI vs. recovered by means of univariate *t* tests. Then, PLS–DA analysis of each phenotype (hepatocellular DILI, cholestatic DILI and recovered patients) vs. the rest was carried out using the complete set of features. Finally, results from the selected models were integrated into a ternary plot.

**Fig. 2:**
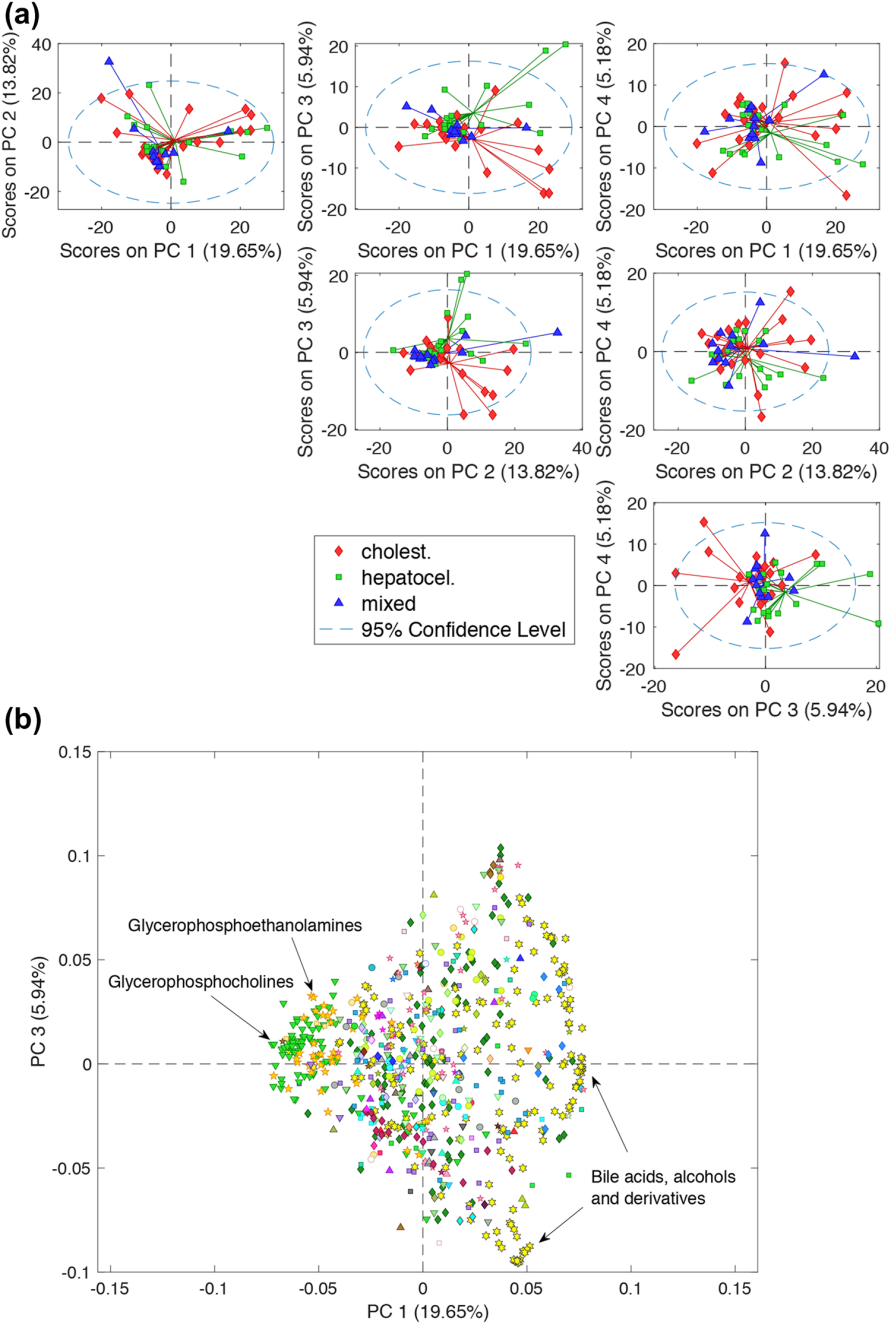
Pairwise combinations of the PC1–PC4 scores (**a**) and PC3 vs. PC4 loadings plot (**b**) from a PCs model build from the autoscaled LC–MS profiles of the DILI samples collected at diagnosis

PCA was used for the explorative analysis of the LC–MS profiles of the samples collected most closely to the diagnosis. Figure 2a shows pairwise combinations of the scores from a 4 PCs model explaining 44.59% of the variation observed in 51 first samples collected from patients after DILI diagnosis and inclusion in the study. As expected, a high overlap across the three types of DILI was observed, which was likely due to a combination of the effects of different types and degrees of DILI severity, treatments, and high inter-individual variability. Nonetheless, PC3 enabled a partial clustering of patients clinically diagnosed as hepatocellular and cholestatic DILI phenotypes. The loadings plot depicted in Fig. 2b revealed that the partial clustering observed in the PC1 vs. PC3 scores space was mainly associated to changes in the relative levels of glycerophosphoethanolamines and glycerophosphocholines (higher in the mixed DILI group), and bile acids and derivatives (higher in the cholestatic group). On the basis of these emerging evidence, we then run univariate *t* tests among the subgroups of cholestatic and hepatocellular DILI patients, and that of recovered patients to identify metabolic features associated to each of the different subcategories. The analysis identified 167 features associated to both hepatocellular and cholestatic DILI. Besides LC–MS features were identified and selected as discriminant for hepatocellular (*n* = 55) or cholestatic (*n* = 101) DILI against the recovered group. Figure SI3 depicts the number and intersections of the differentially expressed metabolomic features (FDR-corrected *t* test *p* values < 0.05) in the hepatocellular vs. cholestatic DILI, and hepatocellular or cholestatic DILI vs. recovered comparisons. The partial overlap of the metabolic features associated to cholestatic and hepatocellular DILI supported the presence of differences in their metabolic phenotypes that could be wisely exploited for a patient’s DILI phenotype diagnosis.

### Integrative model of DILI phenotypes

**Fig. 3:**
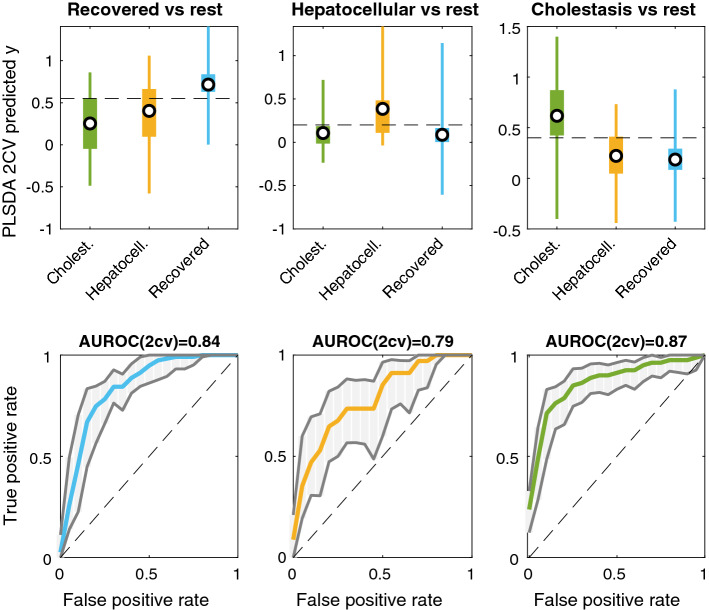
Distribution of predicted “*y*” values obtained for each sample by 2CV in each of the three considered three one-vs-rest models

A set of supervised multivariate PLS–DA models were used to identify differences among the metabolic profiles of cholestatic, hepatocellular, and recovered DILI patients. Subjectwise double cross validation (2CV) (Smit et al. 2007) was selected for the initial assessment of three one-vs-rest models using the complete set of features. The one-vs-rest strategy decompose the original three-classes data set into three binary sub-data sets in which one class is compared with the rest of the classes included in the analysis (Lee and Jemain 2019) (i.e., here, cholestatic vs. the group of hepatocellular and recovered patients, hepatocellular vs. the group of cholestatic and recovered patients, and recovered vs. the group of hepatocellular or cholestatic DILI patients). Figure 3(top) depicts the distribution of PLS predicted *y* values by 2CV for the assignment of the class membership in the three one-vs-rest models. The discrimination among classes was assessed using the areas under the receiver operating characteristic (AUROCs) as figure of merit, and their statistical significance was estimated by permutation testing (100 permutations, *p* values < 0.05) (Lee and Jemain 2019). Results obtained from this analysis support the existence of relevant metabolic differences among the cholestatic, hepatocellular clinical DILI phenotypes and that of recovered patients. Then, three one-vs-rest PLS–DA models were build using again a subjectwise CV for the selection of the number of LVs and the estimation of CV-predicted *y* values for recovered, cholestatic, and hepatocellular DILI samples. These models were then used for the prediction of mixed-type DILI patient’s samples.

**Fig. 4:**
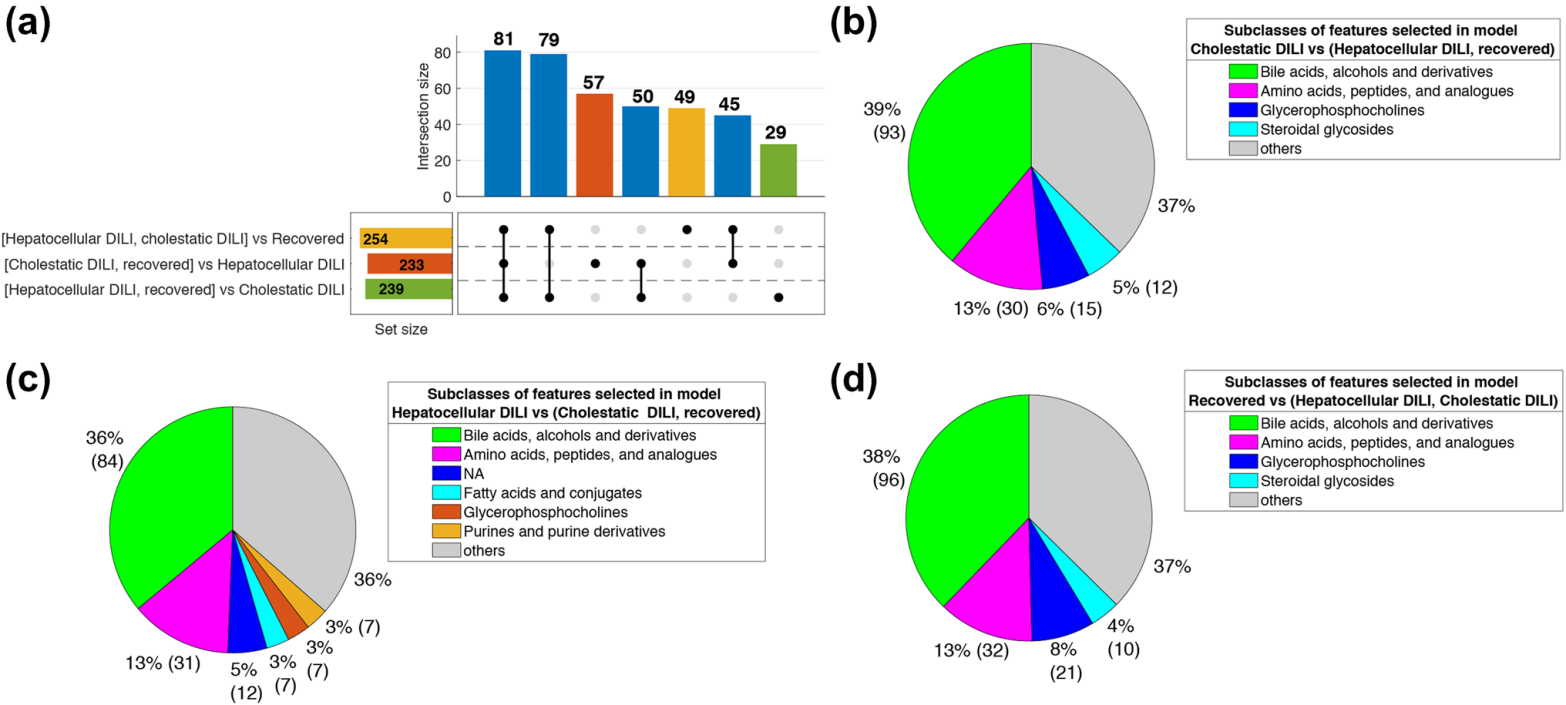
UpSet plot depicting the number and intersections of the features with VIP > 1 in the hepatocellular DILI vs. the group of cholestatic DILI and recovered patients, cholestatic DILI vs. the group of hepatocellular DILI and recovered patients, and recovered vs. the group of hepatocellular and cholestatic DILI patients PLS–DA models. Pie plots show the distributions of the main subclasses of the features selected as discriminant in the three models. Note: NA: features with no subclass included in the HMDB

Figure 4a depicts the number and intersections of the features showing VIP > 1 in the three PLS–DA models. The analysis identified a slightly lower number of features associated to hepatocellular (233) than to cholestatic DILI (239), in agreement with the observed better discrimination of cholestatic samples by PLS–DA. Figure 4b shows that the main subclasses of metabolites selected as discriminant in the cholestatic DILI vs. rest model were bile acids (BAs), amino acids, glycerophosphocholines and steroidal glycosides. It is well known that the normal bile flow out of the liver is disrupted or severely impaired in the course of drug-induced cholestasis. As a consequence of this bile stasis and intrahepatic accumulation of cytotoxic bile acids occur damaging hepatocytes and ductal epithelium cells because of their exposure to high concentrations of BAs. This causes metabolic hepatocyte metabolic impairment as well local inflammatory infiltration leading to hepatocyte cell death. BAs profiles have been recently proposed as DILI biomarkers displaying higher sensitivity than bilirubin for bile excretory abnormalities. A similar distribution of metabolite subclasses was found in the hepatocellular DILI vs. rest model (Fig. 4c) and recovered vs. rest (Fig. 4d). In the latter case, the presence of cholestatic patients with too high serum concentrations of BAs increased the importance of these metabolites in the discrimination. To facilitate the interpretation of the differences among the three classes, a PLS–DA model (2 LVs) was build using the PLS2 algorithm and the set of 390 features selected as discriminant in any of the three previous models, where bile acids, alcohols and derivatives (118), amino acids, peptides and analogues (52), glycerophosphocholines (28), steroidal glycosides (14) and fatty acids and conjugates (13) were the metabolic subclasses with the highest number of features included. The scores plot showed a partial overlap of samples classified as hepatocellular, cholestatic and recovered DILI patients (see Fig. SI4a). Nonetheless, results showed elevated levels of conjugated bile acids (e.g., glycochenodeoxycholic) in the cholestatic group as compared to the hepatocellular and recovered patients, and lower levels of glycerophosphocholines and glycerophosphoethanolamines in DILI compared to recovered patients (see Fig. SI4b). The recovered group also showed slightly higher levels of two steroids and steroid derivatives: deoxycholic acid (a bile acid) and 11-beta-hydroxyandrosterone-3-glucuronide (a steroidal glycoside).

### A novel approach for an easy characterization of DILI sub-phenotypes

**Fig. 5:**
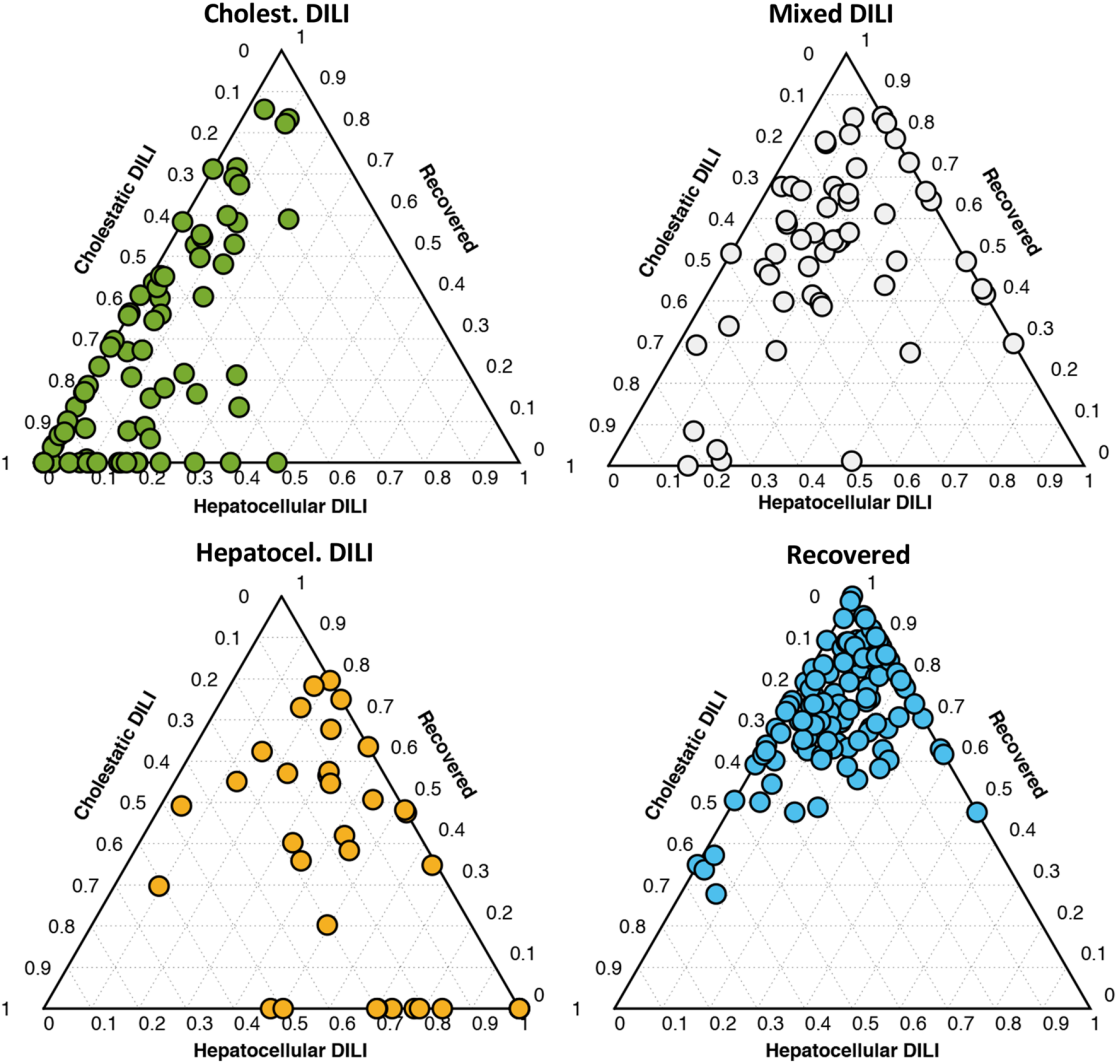
Ternary plots used from the prediction of cholestatic, mixed and hepatocellular DILI samples as well as those from recovered patients using the PLS–DA models hepatocellular DILI vs. the group of cholestatic DILI and recovered patients, cholestatic DILI vs. the group of hepatocellular DILI and recovered patients, and recovered vs. the group of hepatocellular and cholestatic DILI patients

To jointly analyze the results from the three models and to facilitate a visual and straightforward interpretation of a classification outcome, the set of *y* predicted values, as described above, were integrated into a ternary plot. A ternary plot is a two-dimensional graphical representation on three variables that sum to a constant. Under this premise, we hypothesize that the different DILI phenotypes could be expressed to the relative to expression (0–100%) of the features that are typical for cholestasis, hepatocellular or, recovered patients. As PLS–DA *y* predicted values used for sample classification are unbound, *y* predicted values higher than 1 or lower than 0 were replaced by 1 or 0, respectively, and the position within the ternary plot was defined by the relative constrained *y* values. By doing like this, the ternary plot was an equilateral triangle with edges to graphically depict the constrained *y*-predicted values for DILI (PLS–DA model: recovered vs. non-recovered), cholestasis (PLS–DA model: cholestasis vs. non-cholestatic), and hepatocellular (PLS–DA model: hepatocellular vs. non-cholestatic) damages. As displayed in Fig. 5, plotting of the results evidenced a clustering of cholestatic, hepatocellular, and DILI recovered patients in the corners of the ternary diagrams. Recovered patients that displayed neither cholestatic nor hepatocellular metabolomic biomarkers were mostly clustered in the upper corner of the ternary diagram. Samples from purely cholestatic patients with no markers of hepatocellular damage were located in the bottom-left corner area. In the bottom right corner, samples with marked predominant expression of hepatocellular damage and fewer indications of cholestasis were grouped. Patients classified as mixed-type according to the *R*-score were distributed across the ternary plot displaying different degrees of cholestatic and hepatocellular DILI metabolic phenotypes percentages. The distance of the recovered patients to the top corner (i.e., 0% cholestatic, 0% hepatocellular) was also an intuitive indication of how far was the metabolome of the DILI patient from the recovered status, and hence it was of potential utility to estimate the degree of recovery after the DILI episode. An interesting outcome of this representation is that despite patients might have been assigned to the same category, accordingly with the clinical classification of the DILI phenotype based only on clinical biochemical parameters, there is a wide range of differential expression of the characteristic metabolomic biomarkers of that particular phenotype (as expressed as % of belonging to that given category), which would enable a more fine-tuning classification.

### Longitudinal analysis of DILI metabolic sub-phenotypes during treatment

**Fig. 6:**
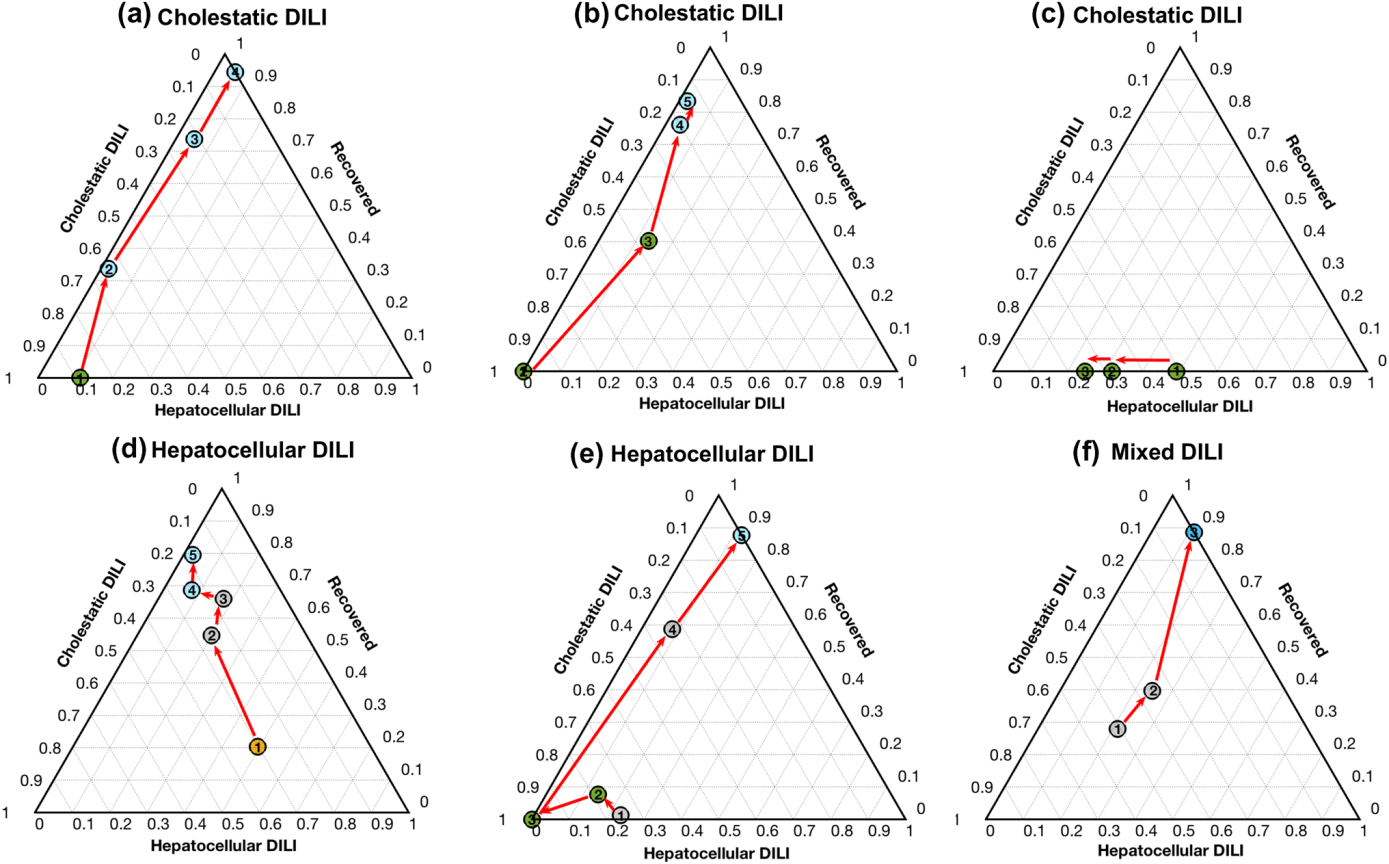
Time course monitoring of four patients initially diagnosed as pure cholestatic, mixed and hepatocellular DILI, towards recovery. Dot colour represents the clinical classification at each point: green: cholestatic DILI; orange: hepatocellular DILI; grey: mixed DILI; blue: recovered patient (color figure online)

The integrated model and graphic representation created were then used to follow-up patients’ metabolome over time after the onset of the DILI event. Figure 6 displays representative examples of the results obtained when time-course monitoring 6 patients. Figure 6a shows a progressive decreasing of a DILI cholestasic profile towards the “recovered” phenotype along the time and, in agreement, the total bilirubin contents between *t*_1_ and *t*_4_ decreased (*t*_1–4_: {12.4, 4.02, 4.02, 1.05}), the *R*-score (*t*_1–4_: {0.64, 0.71, 0.71, 0.57}) and ALT/ALP (*t*_1–4_: {68/277, 32/119, 32/119, 19/87}) values changed accordingly. The evolution of the cholestatic patient depicted in Fig. 6b showed a gradual progression towards recovery after the second sample, in agreement with a significant decrease in the total bilirubin concentrations (*t*_1–5_: {33.6, 21.4, 6.5, 0.6, 0.6}), and ALT/ALP values (*t*_1–5_: {71/212, 49/254, 118/156, 16/102, 12/80}). The patient depicted in Fig. 6c, remained in the cholestasis corner not showing any progression towards the recovered status (ALT/ALP *t*_1–3_: {326/1617, 225/1229, 123/919}). Figure 6d shows the change in the metabolomic profile of a hepatocellular DILI patient towards recovery (*R*-score *t*_1–5_: {32.0, 4.3, 1.5, 1.1, 1.1}; total bilirubin *t*_1–5_: {25.9, 7.6, 4.5, 2.8, 0.7}; ALT/ALP *t*_1–5_: {2293/188, 194/117, 70/119, 44/101, 47/105}). However, the clinical situation of the patient depicted in Fig. 6e did not improve during the follow-up and his metabolome showed a hepatocellular phenotype again, in clinical agreement, with elevated ALT/ALP values (*t*_1–3_: {1298/161, 1188/179, 884/127}). The relative position of the first samples of patients depicted in Fig. 6d, e correlated with the higher severity of the DILI observed in the latter patient and the higher levels of total bilirubin contents in the first one. Finally, the patient depicted in Fig. 5f, that belonged to the mixed-type phenotype, although recovering over time, maintained all time the features of a mixed-type injury (ALT/ALP *t*_1–5_: {229/228, 138/214, 117/168, 106/88, 34/66}).

As disclosed, this novel graphic approach, displays features that enable a better description of the phenotype status of the patient, and it could be used to monitor the progression of DILI patients to their recovery and even anticipate its clinical evolution.

This research is a fact-finding exercise that, despite the limited number of samples studied, has provided a well sustained and innovative background information, for the study of hepatotoxicity and DILI classification approach. A systematic application for the monitoring of a larger set of patient’s data will help to reinforce its relevance, to disclose potential biases and confounding sources. Metabolite annotation by MS/MS is currently limited and further targeted analysis of selected metabolite classes such as phosphatidylcholines (PCs), lysophosphatidylcholines (LysoPCs), and bile acids (primary, secondary, conjugated) will add more confidence to the model and stimulate its use in a routine clinical setting.

## Conclusions

The analysis and relationships between liver endo- and exo-metabolome can provide inherent high-level information on the type and severity of the liver toxicity and damage after a chemical insult, and is, therefore, conceivable that by the use of such information and proper bioinformatics modeling of the recorded changes, be possible a more accurate interpretation of drug liver toxicity and a precise DILI diagnosis, monitoring the progression of the disease and anticipating its evolution. We have exhaustively analyzed the liver exo-metbolome present in the sera of patients undergoing a DILI event, and extracted the ground toxicity information that distinguish the different DILI phenotypes and the “recovered”, clinically asymptomatic, status. Serum bile acid profiles including primary, secondary, conjugated, and non‐conjugated bile acids, combined with glycerophospholipids (glycerophosphocholines, glycerophosphoethanolamines) were the metabolites that best discriminated among the cholestatic and hepatocellular DILI phenotypes from the onset of the disease until recovery. We found the information generated by the bioinformatics model, useful and complementary to that provided by the *R*-score for a more precise and accurate monitoring of the DILI event. The strategy used in our work, with an appropriate treatment and integration of the data recorded, and the presentation of such degree of data information in ternary plots enabled a visual and straightforward classification of DILI phenotypes of patients. Moreover, the ternary diagrams facilitated the monitoring of the disease progress towards recovery. The results of this research and the building up of a quantitative model reinforced the view that metabolomics can provide a detailed insight into the study of liver drug toxicity and the different DILI phenotypic patterns and a unique dynamic readout of DILI progression during recovery complementary to that provided by standard clinical biochemistry biomarkers. Additional efforts are required to reinforce the clinical value of the metabolic patterns observed, using other complementary approaches (e.g., lipidomics) as well its clinical validation using quantitative approaches, to be undertaken in future clinical studies.

## Supplementary Information

Below is the link to the electronic supplementary material.

Supplementary file1. Fig. SI1 (Top) Scores of two-components principal component analysis (PCA) models explaining 32% and 43% of the data variation in the ESI+ (left) and ESI− (right) data sets, respectively. (Bottom) Projection of the samples analysed in both, batches 1 and 2 used for the correction of between-batch effects, in the PCA models depicted on top. (PDF 523 kb)

Supplementary file2. Fig. SI2 (Left) Distribution of LC−MS annotated features and main metabolic classes retained after data pre-processing. (Right) Pie plot representing the relative percentages of annotated features of the main classes of metabolites. (PDF 127 kb)

Supplementary file3. Fig. SI3 UpSet plot depicting the number and intersections of the differentially expressed features in the hepatocellular vs. cholestatic, and hepatocellular or cholestatic vs. recovered comparisons (*t* test analysis, unequal variances, *p* value threshold: 0.05). (PDF 34 kb)

Supplementary file4. Fig. SI4 PLS–DA LV1 vs. LV2 scores plot (**a**) and loadings plot (**b**) from a model build for the discrimination of hepatocellular, cholestatic and recovered patients using a set of 538 features selected as discriminant in at least one of the hepatocellular vs. rest, cholestatic vs. rest, and recovered vs. rest PLS–DA models (see the text for details) (PDF 142 kb)

## Data Availability

The data sets generated during and/or analysed during the current study are available from the corresponding author on reasonable request, including the metadata, and the curated and annotated peak table as MATLAB.mat files.

## Code availability

Non-applicable.

## References

[CR1] Abajo FJD, Montero D, Madurga M, Rodríguez LAG (2004). Acute and clinically relevant drug-induced liver injury: a population based case-control study. Br J Clin Pharmacol.

[CR2] Aithal GP, Watkins PB, Andrade RJ, Larrey D, Molokhia M, Takikawa H, Hunt CM, Wilke RA, Avigan M, Kaplowitz N, Bjornsson E, Daly AK (2011). Case definition and phenotype standardization in drug-induced liver injury. Clin Pharmacol Ther.

[CR3] Andrade RJ, Chalasani N, Björnsson ES, Suzuki A, Kullak-Ublick GA, Watkins PB, Devarbhavi H, Merz M, Lucena MI, Kaplowitz N, Aithal GP (2019). Drug-induced liver injury. Nat Rev Dis Primers.

[CR4] Araújo AM, Carvalho M, Carvalho F, Bastos MDL, Guedes de Pinho P (2017). Metabolomic approaches in the discovery of potential urinary biomarkers of drug-induced liver injury (DILI). Crit Rev Toxicol.

[CR5] Benichou C, Danan G, Flahault A (1993). Causality assessment of adverse reactions to drugs—II. An original model for validation of drug causality assessment methods: case reports with positive rechallenge. J Clin Epidemiol.

[CR6] Benjamini Y, Hochberg Y (1995). Controlling the false discovery rate: a practical and powerful approach to multiple testing. J R Stat Soc Ser B Methodol.

[CR7] Björnsson ES, Bergmann OM, Björnsson HK, Kvaran RB, Olafsson S (2013). Incidence, presentation, and outcomes in patients with drug-induced liver injury in the general population of Iceland. Gastroenterology.

[CR8] Broadhurst D, Goodacre R, Reinke SN, Kuligowski J, Wilson ID, Lewis MR, Dunn WB (2018). Guidelines and considerations for the use of system suitability and quality control samples in mass spectrometry assays applied in untargeted clinical metabolomic studies. Metabolomics.

[CR9] Cañaveras JCG, Castell JV, Donato MT, Lahoz A (2016). A metabolomics cell-based approach for anticipating and investigating drug-induced liver injury. Sci Rep.

[CR10] Chang C-C, Lin C-J (2011). LIBSVM: a library for support vector machines. ACM Trans Intell Syst Technol TIST.

[CR11] Danan G, Benichou C (1993). Causality assessment of adverse reactions to drugs—I. A novel method based on the conclusions of international consensus meetings: application to drug-induced liver injuries. J Clin Epidemiol.

[CR12] Devarbhavi H (2012). An update on drug-induced liver injury. J Clin Exp Hepatol.

[CR13] The Food an Drug Administration; Federal Register (2009) Guidance for industry on drug-induced liver injury: premarketing clinical evaluation; availability. In: Federal Register. https://www.federalregister.gov/documents/2009/07/30/E9-18135/guidance-for-industry-on-drug-induced-liver-injury-premarketing-clinical-evaluation-availability. Accessed 20 Aug 2018

[CR14] García-Cortés M, Stephens C, Lucena MI, Fernández-Castañer A, Andrade RJ (2011). Causality assessment methods in drug induced liver injury: strengths and weaknesses. J Hepatol.

[CR27] García-Cañaveras JCG (2015) Metabolomics as a tool for the study of drug-induced hepatotoxicity. PhD Thesis, University of Valencia 2015. http://mobiroderic.uv.es/handle/10550/42776

[CR15] Hutchins PD, Russell JD, Coon JJ (2018). LipiDex: an integrated software package for high-confidence lipid identification. Cell Syst.

[CR16] Iruzubieta P, Arias-Loste MT, Barbier-Torres L, Martinez-Chantar ML, Crespo J (2015). The need for biomarkers in diagnosis and prognosis of drug-induced liver disease: does metabolomics have any role?. BioMed Res Int.

[CR17] Kind T, Liu K-H, Lee DY, DeFelice B, Meissen JK, Fiehn O (2013). LipidBlast in silico tandem mass spectrometry database for lipid identification. Nat Methods.

[CR18] Kuligowski J, Sánchez-Illana Á, Sanjuán-Herráez D, Vento M, Quintás G (2015). Intra-batch effect correction in liquid chromatography-mass spectrometry using quality control samples and support vector regression (QC-SVRC). Analyst.

[CR19] Larrey D (2000). Drug-induced liver diseases. J Hepatol.

[CR20] Lee LC, Jemain AA (2019). Predictive modelling of colossal ATR-FTIR spectral data using PLS-DA: empirical differences between PLS1-DA and PLS2-DA algorithms. Analyst.

[CR21] Lin N-H, Yang H-W, Su Y-J, Chang C-W (2019). Herb induced liver injury after using herbal medicine. Medicine.

[CR22] Maria VA, Victorino RM (1997). Development and validation of a clinical scale for the diagnosis of drug-induced hepatitis. Hepatology.

[CR23] Mattes W, Davis K, Fabian E, Greenhaw J, Herold M, Looser R, Mellert W, Groeters S, Marxfeld H, Moeller N, Montoya-Parra G, Prokoudine A, van Ravenzwaay B, Strauss V, Walk T, Kamp H (2014). Detection of hepatotoxicity potential with metabolite profiling (metabolomics) of rat plasma. Toxicol Lett.

[CR24] McGill MR, Jaeschke H (2019). Biomarkers of drug-induced liver injury. Adv Pharmacol.

[CR25] O’Connell TM, Watkins PB (2010). The application of metabonomics to predict drug-induced liver injury. Clin Pharmacol Ther.

[CR26] Robles-Díaz M, Medina-Caliz I, Stephens C, Andrade RJ, Lucena MI (2016). Biomarkers in DILI: one more step forward. Front Pharmacol.

[CR29] Russmann S, Kullak-Ublick GA, Grattagliano I (2009). Current concepts of mechanisms in drug-induced hepatotoxicity. Curr Med Chem.

[CR30] Sánchez-Illana Á, Pérez-Guaita D, Cuesta-García D, Sanjuan-Herráez JD, Vento M, Ruiz-Cerdá JL, Quintás G, Kuligowski J (2018). Model selection for within-batch effect correction in UPLC-MS metabolomics using quality control - support vector regression. Anal Chim Acta.

[CR31] Sánchez-Illana Á, Piñeiro-Ramos JD, Sanjuan-Herráez JD, Vento M, Quintás G, Kuligowski J (2018). Evaluation of batch effect elimination using quality control replicates in LC-MS metabolite profiling. Anal Chim Acta.

[CR32] Sgro C, Clinard F, Ouazir K, Chanay H, Allard C, Guilleminet C, Lenoir C, Lemoine A, Hillon P (2002). Incidence of drug-induced hepatic injuries: a French population-based study. Hepatology.

[CR33] Smit S, van Breemen MJ, Hoefsloot HCJ, Smilde AK, Aerts JMFG, de Koster CG (2007). Assessing the statistical validity of proteomics based biomarkers. Anal Chim Acta.

[CR34] Smith CA, Want EJ, O’Maille G, Abagyan R, Siuzdak G (2006). XCMS: processing mass spectrometry data for metabolite profiling using nonlinear peak alignment, matching, and identification. Anal Chem.

[CR35] Tang W, Xu Q (2014) Chapter 11: Metabolomics investigations of drug-induced hepatotoxicity. In: Grootveld M (ed) Issues in toxicology 21; Metabolic profiling: disease and Xenobiotics. The Chemical Society, London 2015, pp 323–356. 10.1039/9781849735162

[CR36] Ten-Doménech I, Martínez-Sena T, Moreno-Torres M, Sanjuan-Herráez JD, Castell JV, Parra-Llorca A, Vento M, Quintás G, Kuligowski J (2020). Comparing targeted vs. untargeted MS2 data-dependent acquisition for peak annotation in LC-MS metabolomics. Metabolites.

[CR37] Watkins PB (2013). Biomarkers for drug-induced liver injury. Drug Induc Liver Dis.

[CR38] Wold S, Sjöström M, Eriksson L (2001). PLS-regression: a basic tool of chemometrics. Chemom Intell Lab Syst.

[CR39] Yang J, Yang L, Yu S, Liu J, Zuo J, Chen W, Duan W, Zheng Q, Xu X, Li J, Zhang J, Xu J, Sun L, Yang X, Xiong L, Yi D, Wang L, Liu Q, Ge S, Ren J (2014). Transcatheter versus surgical closure of perimembranous ventricular septal defects in children: a randomized controlled trial. J Am Coll Cardiol.

[CR40] Zimmerman HJ (2000). Drug-induced liver disease. Clin Liver Dis.

