# Supplementary figures and images for: Metabolomic analysis to discriminate drug-induced liver injury (DILI) phenotypes
Source: Arch Toxicol. 2021 Jul 17;95(9):3049–62. doi: 10.1007/s00204-021-03114-z (PMC8380240; doi:10.1007/s00204-021-03114-z)

Figure S11

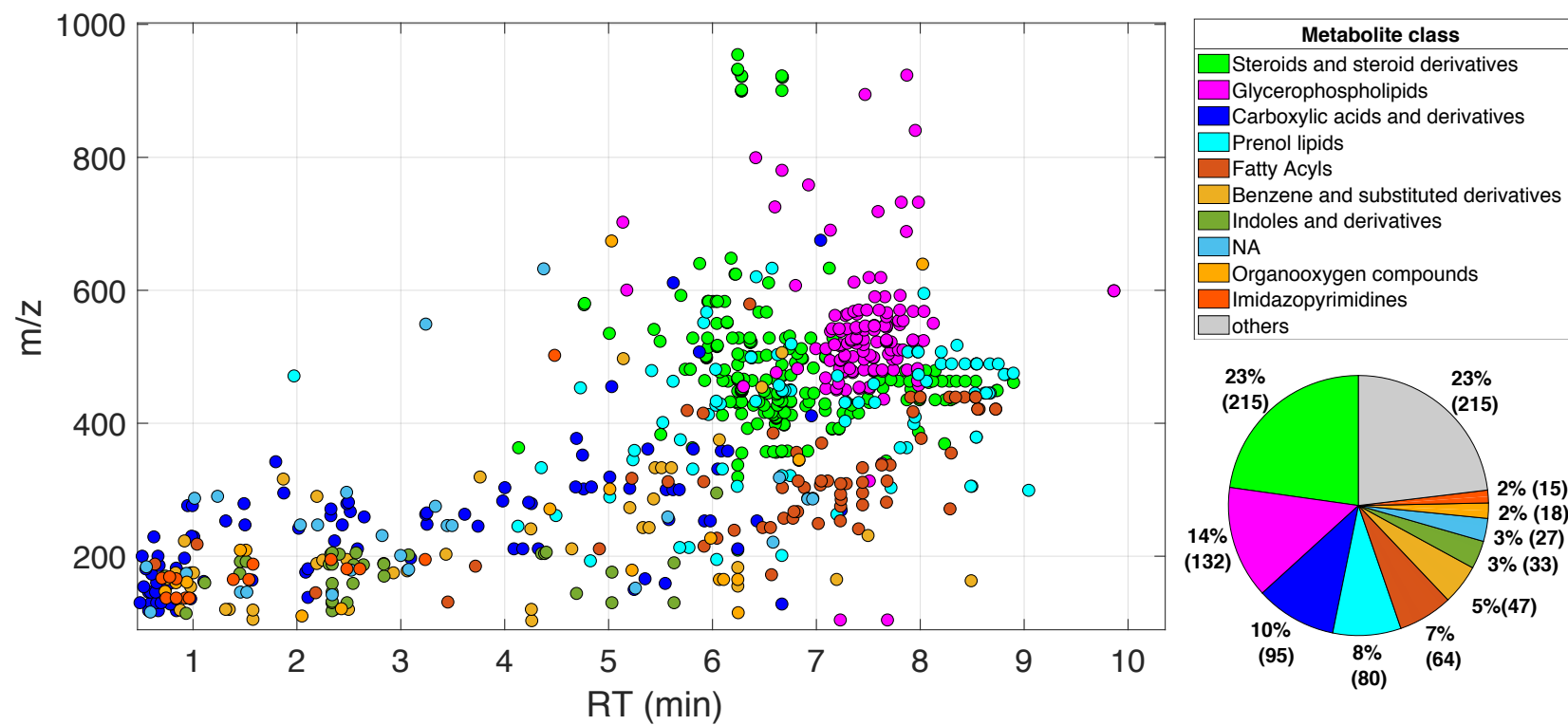

Supplement: Supplementary file 1 — Supplementary file1. Fig. SI1 (Top) Scores of two-components principal component analysis (PCA) models explaining 32% and 43% of the data variation in the ESI+ (left) and ESI− (right) data sets, respectively. (Bottom) Projection of the samples analysed in both, batches 1 and 2 used for the correction of between-batch effects, in the PCA models depicted on top. (PDF 523 kb) [file 204_2021_3114_MOESM1_ESM.pdf]

Figure S12

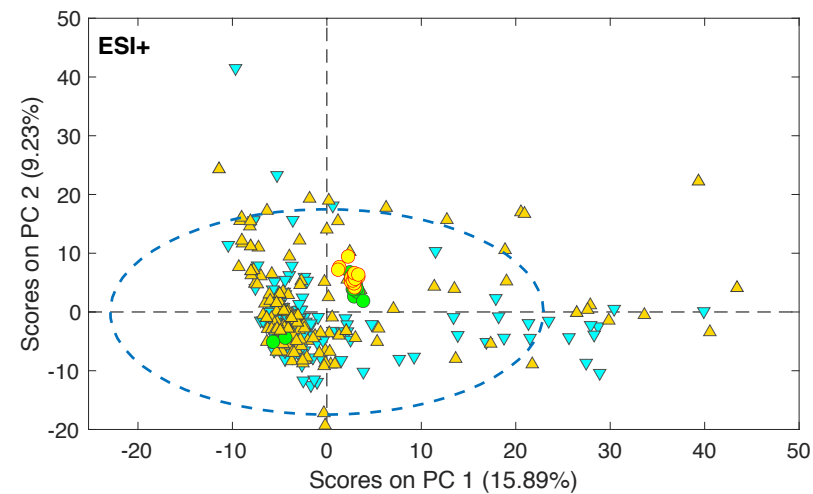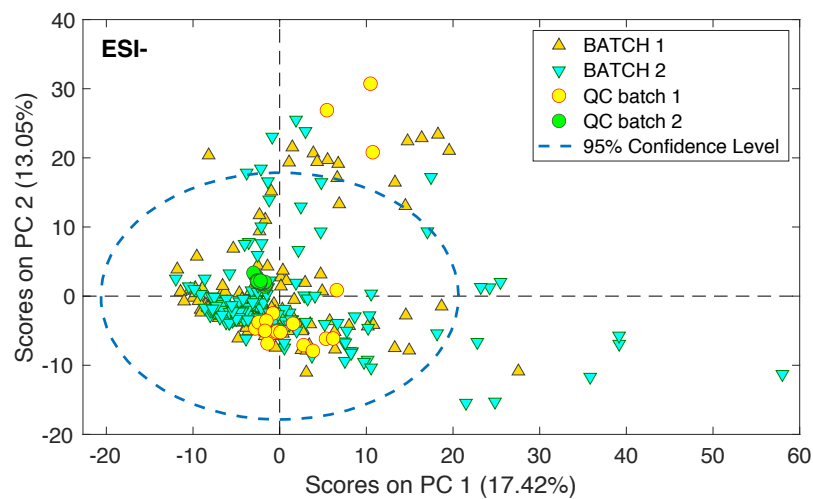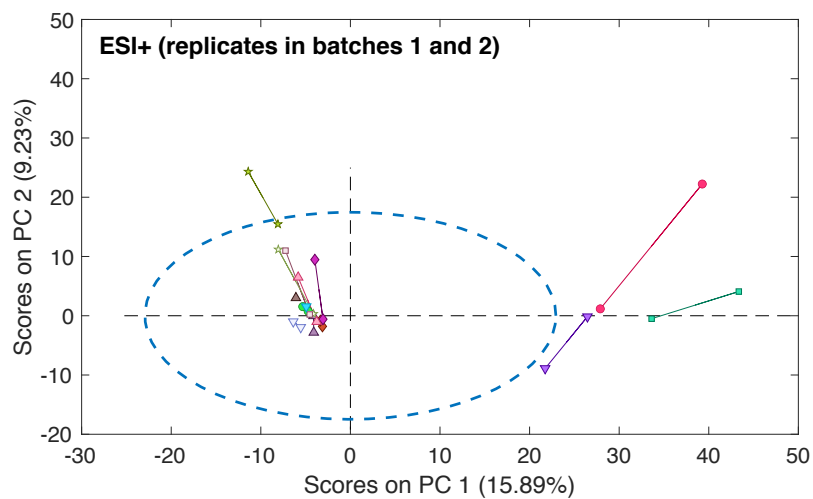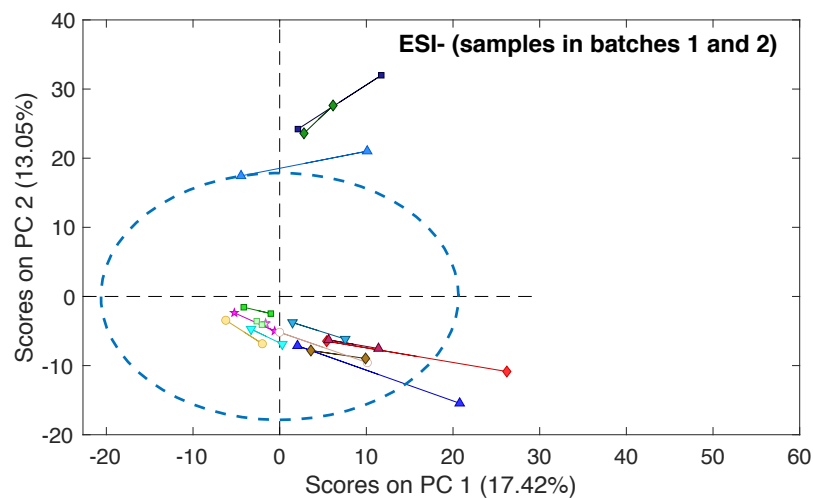

Supplement: Supplementary file 2 — Supplementary file2. Fig. SI2 (Left) Distribution of LC−MS annotated features and main metabolic classes retained after data pre-processing. (Right) Pie plot representing the relative percentages of annotated features of the main classes of metabolites. (PDF 127 kb) [file 204_2021_3114_MOESM2_ESM.pdf]

Figure S13

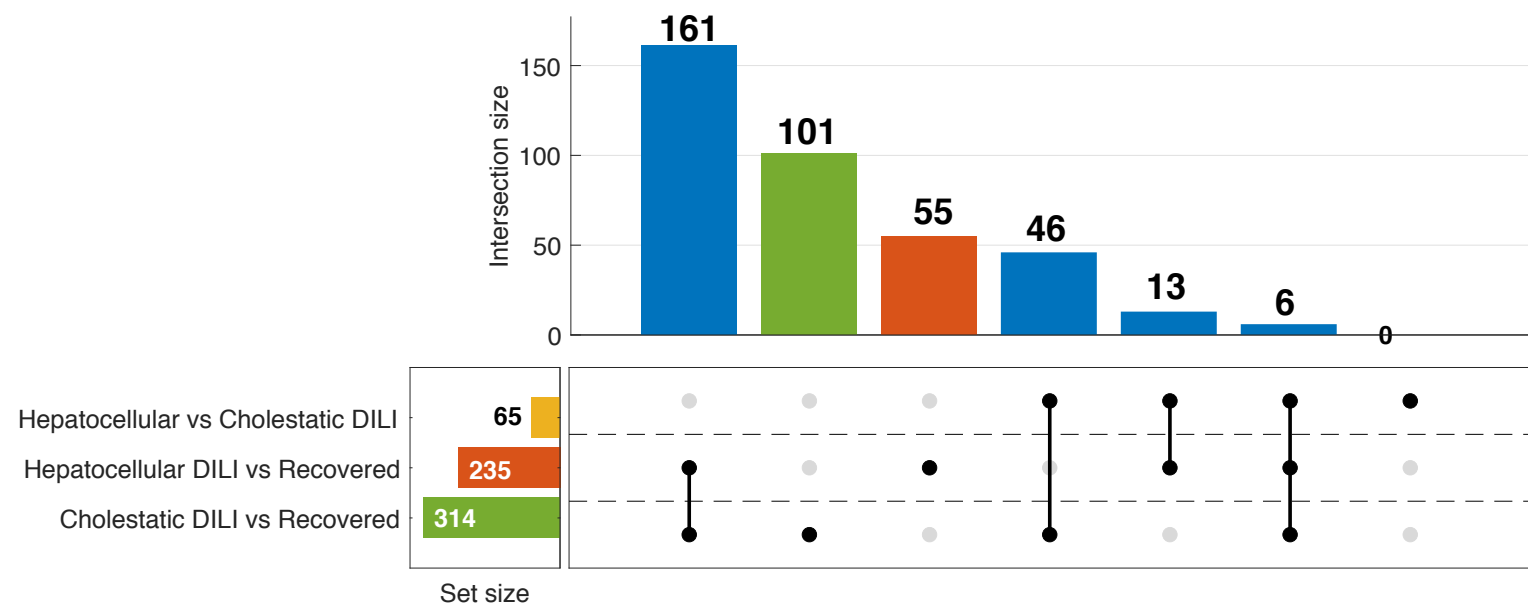

Supplement: Supplementary file 3 — Supplementary file3. Fig. SI3 UpSet plot depicting the number and intersections of the differentially expressed features in the hepatocellular vs. cholestatic, and hepatocellular or cholestatic vs. recovered comparisons (t test analysis, unequal variances, p value threshold: 0.05). (PDF 34 kb) [file 204_2021_3114_MOESM3_ESM.pdf]

Figure S14

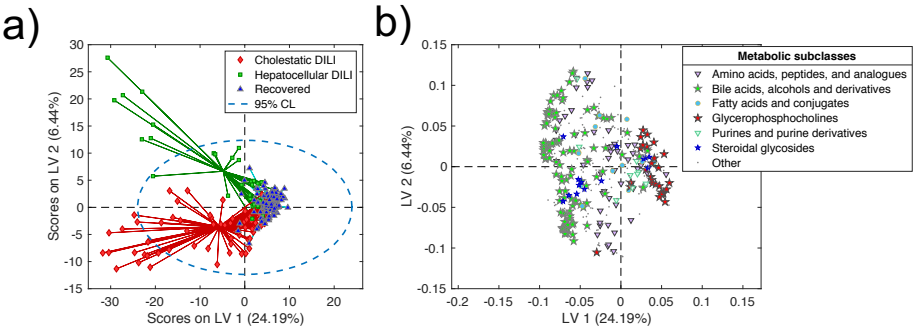

Supplement: Supplementary file 4 — Supplementary file4. Fig. SI4 PLS–DA LV1 vs. LV2 scores plot (a) and loadings plot (b) from a model build for the discrimination of hepatocellular, cholestatic and recovered patients using a set of 538 features selected as discriminant in at least one of the hepatocellular vs. rest, cholestatic vs. rest, and recovered vs. rest PLS–DA models (see the text for details) (PDF 142 kb) [file 204_2021_3114_MOESM4_ESM.pdf]
